# Cone-Beam Breast Computed Tomography: Time for a New Paradigm in Breast Imaging

**DOI:** 10.3390/jcm10215135

**Published:** 2021-10-31

**Authors:** Avice M. O’Connell, Thomas J. Marini, Daniel T. Kawakyu-O’Connor

**Affiliations:** Department of Imaging Sciences, University of Rochester Medical Center, 601 Elmwood Ave, Rochester, NY 14642, USA; rochesterradiology2021@gmail.com (T.J.M.); Daniel_Oconnor@URMC.rochester.edu (D.T.K.-O.)

**Keywords:** breast cancer, cone-beam breast computed tomography (CBBCT), mammography

## Abstract

It is time to reconsider how we image the breast. Although the breast is a 3D structure, we have traditionally used 2D mammography to perform screening and diagnostic imaging. Mammography has been continuously modified and improved, most recently with tomosynthesis and contrast mammography, but it is still using modifications of compression 2D mammography. It is time to consider 3D imaging for this 3D structure. Cone-beam breast computed tomography (CBBCT) is a revolutionary modality that will assist in overcoming the limitations of current imaging for dense breast tissue and overlapping structures. It also allows easy administration of contrast material for functional imaging. With a radiation dose on par with diagnostic mammography, rapid 10 s acquisition, no breast compression, and true high-resolution isotropic imaging, CBBCT has the potential to usher in a new era in breast imaging. These advantages could translate into lower morbidity and mortality from breast cancer.

## 1. The Current State of Breast Imaging

Millions of new cases of breast cancer occur every year worldwide with about 42,000 deaths from breast cancer in the United States and 500,000 deaths worldwide [1,2]. Can we not do better?

**Figure 1:**
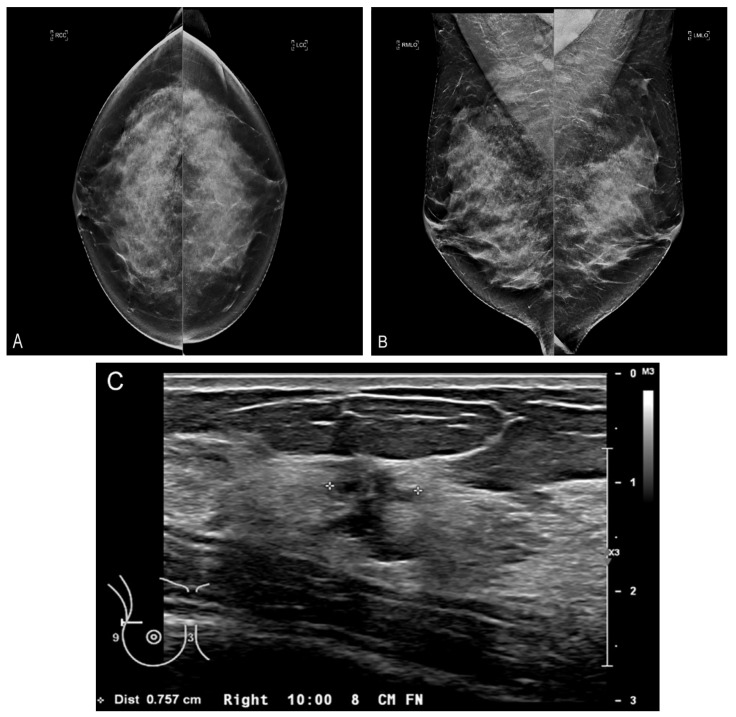
Limitation of mammography for dense breasts. Bilateral craniocaudal (CC) (**A**) and bilateral mediolateral oblique (MLO) (**B**) mammograms showing extremely dense breasts (ACR density category d) [7]. The mammogram is negative (Breast Imaging Reporting and Data System (BI-RADS) 1). (**C**) Fortunately, the patient had a screening ultrasound shortly thereafter showing a 1 cm cancer at the 10 o’clock position in the right breast. The mammogram was a false negative. The patient came to no harm since the ultrasound was performed within a short period of time. The cancer was grade I invasive ductal carcinoma with negative nodes. Survival after treatment is near 100% [8].

Here is the problem: one in eight women in the United States will get breast cancer in their lifetime. Some cancers will be detected on screening. Many will not. Even if a woman gets regular screening and has no known risk factors, her cancer may still be missed [3,4]. Additionally, we keep telling women that mammography saves lives, but we are less willing to admit the low sensitivity and specificity of mammography, especially in dense breasts (Figure 1) [5,6]. Why is this still happening? We need to do better. We owe it to the women who place their trust in us.

There is no perfect test. For average risk women, the best widely available screening modality around the world today is mammography. Mammography requires two separate views with uncomfortable compression, on average approximately 20 pounds or 90 Newtons [9,10]. Additionally, when abnormalities are visualized on a screening exam they can never be exactly co-registered on the two standard views because they are two separately acquired views (craniocaudal (CC) and mediolateral oblique (MLO)), which are not orthogonal. In mammography, there is the problem of false positive findings due to overlap and breast density but also too many false negatives, especially in dense breasts [11]. Overall mammography sensitivity is quoted from 75% to 90% and specificity from 90% to 95% [12]. However, the sensitivity for mammography is dramatically lower in dense breasts [5]. Think of looking for a snowman in a snowstorm, or even more challenging, a snowball in a snowstorm. This is the situation when we try to visualize a white cancer in a field of dense white breast parenchyma. Because glandular tissue and malignant masses have similar density characteristics, mammography cannot distinguish between the two. The diagnostic challenge posed by dense breasts is compounded by the increased risk of cancer associated with increased breast density. It is known that women with mammographically dense breasts have a relative risk of cancer 4–6 times that of women with fatty breasts [13]. With greater than 50% of women categorized as heterogeneously or extremely dense in their 40’s and 50’s (ACR density categories c or d), this is a non-trivial problem [7,14]. This places additional burdens on imaging; not only is the sensitivity of mammography less than 50% in extremely dense breasts, but there is the additional burden of more cancer.

The latest improvement to mammography is called tomosynthesis (approved by the FDA in 2011), which provides a reconstructed pseudo-3D image from multiple 2D images taken over different angles [15]. Tomosynthesis offers marginally increased sensitivity and specificity compared to mammography [16]. Even with the advantages of tomosynthesis, dense breasts present a persistent diagnostic challenge. Thankfully, supplemental screening with other imaging modalities is available to women with extremely dense breasts, family history of cancer, or other risk factors [17]. Supplemental screening for breast cancer with magnetic resonance imaging (MRI) is usually reserved for women at high risk (>20% lifetime risk) [18]. Contrast-enhanced MRI comes close to 100% in sensitivity for invasive disease, but its specificity is slightly less [19]. It is not practical or economical to screen every woman annually with MRI. MRI is also not available to many women due to various factors including availability, access, and cost [4,20,21,22,23]. However, even contrast-enhanced MRI has several limitations. In addition to high cost, limited availability, claustrophobia, and issues with the contrast agent gadolinium, the spatial resolution of images on a 1.5 T magnet is only around 1 mm at best [24]. Intermediate risk women (12–20% lifetime risk) and those with extremely dense breast density may be offered supplemental screening with breast ultrasound [25,26]. Unfortunately, not all women have access to or even awareness of the value and availability of this additional imaging [27,28].

**Figure 2:**
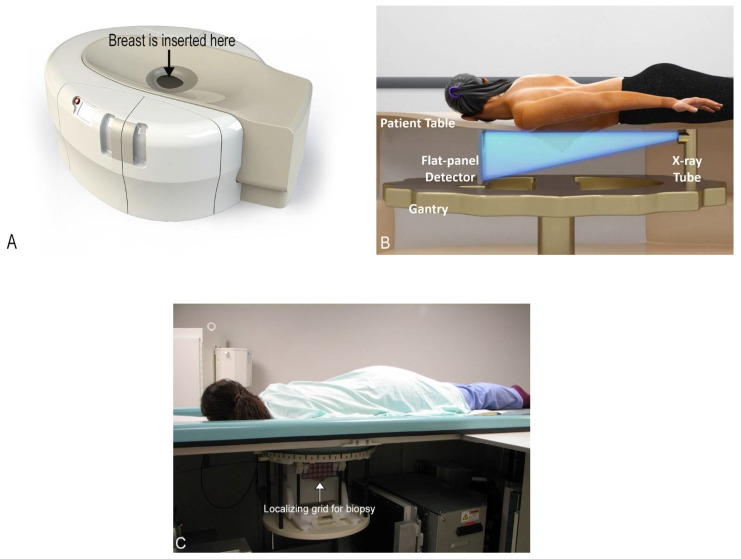
Cone-beam breast computed tomography (CBBCT) basics. (**A**) Picture of a CBBCT machine with the area that the patient inserts her breast labeled. (**B**) Labeled schematic of a CBBCT machine. The patient is positioned prone in the scanner and one breast is imaged at a time. (**C**) Photo of a patient in position for biopsy with localizing grid for biopsy labeled.

We need to rethink the whole situation. A new paradigm in breast imaging is needed to address the fundamental issues presented above. If you knew nothing about the way breast imaging is performed today, and you were tasked with devising a way to image a woman’s breast comfortably and accurately, you would almost certainly not start by distorting and compressing this highly sensitive structure multiple times, causing considerable pain to most women. With today’s imaging, if you see something unusual on the first two mammographic images (CC and MLO), you take many more views. However, with your ideal modality, you would utilize 3D imaging from the start and image the entire breast once, without compression or distortion, and you would do it with perfect 3D isotropic resolution. Then, after reconstruction of the initial image in all three planes (transverse, sagittal, and coronal), all the information needed can be retrieved. This modality would also have the availability of functional imaging with contrast enhancement without having undue radiation exposure. This ideal modality is actually currently a reality with cone-beam breast computed tomography (CBBCT) (Figure 2 and Supplementary Video S1) [29].

## 2. Cone-Beam Breast Computed Tomography

CBBCT was developed with the full knowledge of the many limitations of the current accepted screening modalities available around the world [29,30,31,32,33,34]. The first unit to receive FDA approval for diagnostic use in the United States in 2015 was developed by the Koning Corporation (Norcross, GA, USA). In this system, the patient positions herself prone in the machine one breast at a time, inserting her own breast into the opening in the table, thereby placing the breast in the image field (Figure 2). No compression is used which vastly improves comfort. There is also no intrusive handling of the breasts, which is a completely new consideration of privacy and cultural reservations not previously addressed with current technologies. The technologist checks positioning, making any necessary minor adjustments and then takes one 360-degree image in a period of 10 s. The patient holds still, without a breath hold required. The second breast is imaged in the same way. Efficiency or speed of imaging is another advantage of CBBCT. Standard MRI takes at least 30 min, but with CBBCT, breasts (including the chest wall and axilla) are imaged one side at a time using a single 10 s 360-degree sweep.

CBBCT is not yet approved for screening in the United States at the time of this writing. Its current application is for diagnostic use, which includes all the indications for conventional diagnostic mammography including recall from screening and evaluation of palpable abnormalities. It is also useful for women who cannot tolerate conventional mammography for various reasons. As of May 2020, CBBCT also has its own CPT codes [35]. The device also has biopsy capability for findings only seen or best seen by CBBCT (Figure 2). This is analogous to the technique used for prone stereotactic biopsies and MRI biopsies. An additional benefit to CT biopsy is the potential to have excellent evaluation of the surrounding target vasculature. The radiation dose for CBBCT is well within the accepted range for diagnostic imaging and is sometimes less than that of diagnostic mammography [32,36]. For reference, one study found an average mean glandular dose of 13.9 mGy from CBBCT (range 5.7–27.8 mGy) and an average mean glandular dose of 12.4 mGy from mammography (range 2.6–31.6 mGy) [36]. However, this dose range still may preclude its use in routine screening.

**Figure 3:**
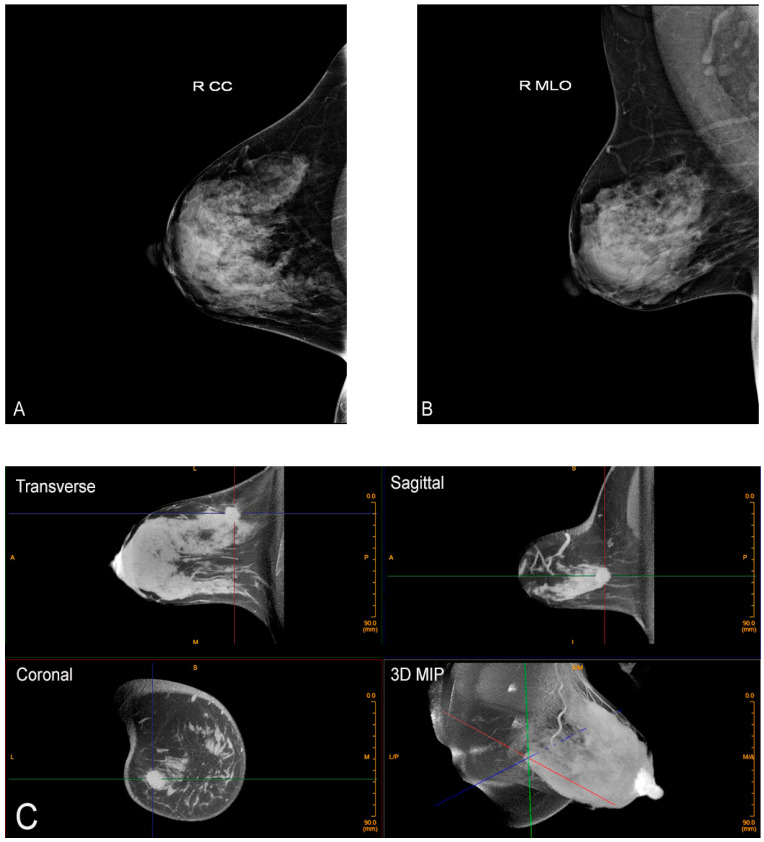
Advantage of CBBCT over mammography for imaging dense breast tissue. Bilateral CC (**A**) and bilateral MLO (**B**) views demonstrate dense breasts without focal findings consistent with a negative or BI-RADS 1 mammogram. (**C**) Post-contrast CBBCT demonstrates a mass denoted by guidelines that is easily recognized consistent with cancer.

Additional advantages of this new technology are many. Because the breast is imaged prone without compression or distortion, there is no overlapping tissue, thereby reducing the likelihood of false positives (a common cause of anxiety provoking recalls for additional imaging (Breast Imaging Reporting and Data System (BI-RADS) 0), which also has benefits for healthcare costs and decreasing unnecessary biopsies. However, perhaps the greatest benefit is CBBCT’s potential increased sensitivity for cancers in dense breasts over mammography, which, as previously discussed, is fundamentally ill-equipped for dense breast evaluation. CBBCT provides a more sensitive evaluation for dense breasts, which is critical as, again, these are the patients at greatest risk for cancer (Figure 3) [33]. Better evaluation of dense breast tissue ultimately would equate to earlier cancer detection, less morbidity, and potentially greater cancer survival.

**Figure 4:**
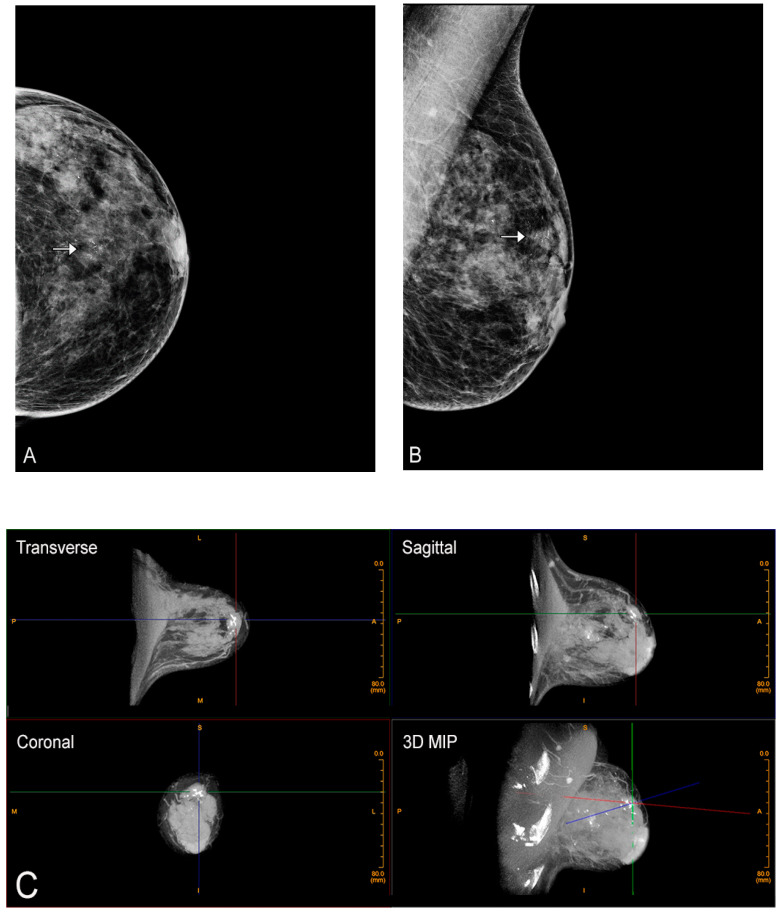

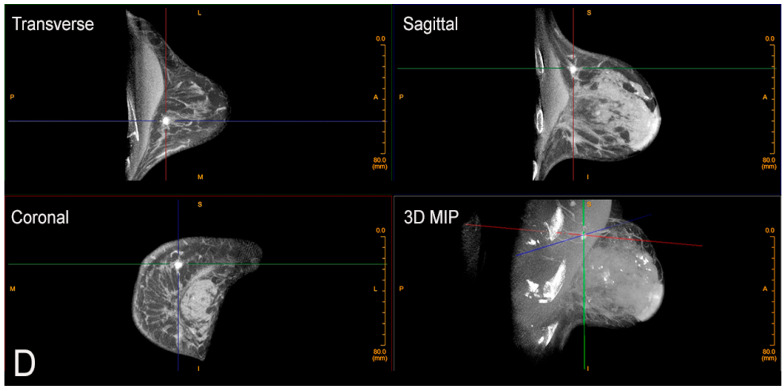
Calcifications on CBBCT. Left CC (**A**) and MLO (**B**) views demonstrate pleomorphic micro-calcifications (arrow). (**C**) Unenhanced CBBCT images showing calcifications, which are marked with grid lines. (**D**) Contrast-enhanced CBBCT in the same patient showed an incidental mass marked with grid lines.

This technique also produces true isotropic 3D imaging without a breath hold necessary. Isotropic imaging refers to an imaging process with the same spatial resolution in the X, Y, and Z planes, resulting in a base imaging unit (voxel) equivalent to a perfect cube. The standard imaging unit in the Koning CBBCT machine is 0.273 mm in the X, Y, and Z planes (significantly superior to MRI, which is around 1 mm for a 1.5 T magnet) [24,33]. A high-resolution mode for calcifications can have spatial resolution of 0.122 mm in the X, Y, and Z planes. Cancer detection requires this excellent spatial resolution, especially for the evaluation of micro-calcifications, which are present in about 55% of non-palpable cancers [37]. Most suspicious calcifications are in the range of 100 microns (0.1 mm) [38]. While MRI is not always able to add to the diagnostic work-up of microcalcifications, CBBCT does show calcium with adequate resolution (Figure 4) [39].

An additional fundamental advantage of isotropic imaging is that accurate imaging reconstructions in all planes (transverse, sagittal, and coronal) can be reconstructed based off the single acquisition without any image distortion. We like to say you can “manipulate the image, not the patient”. Understandably, women much prefer this. The benefits of isotropic imaging include excellent data acquisition, superior display, and greater compliance from the patient. When combined with compression-free imaging of the breast, the result is true anatomical images of the breast free of anatomical or artifactual distortions. This corresponds to better presurgical planning, ability to perform accurate volumetric analysis for treatment response, quantitative estimates of implant rupture, and overall improved diagnostic accuracy. CBBCT has also shown promise in assisting in the evaluation of different breast cancer types based on imaging morphology [40].

Most screening today involves morphological or structural imaging, looking for a mass, asymmetry, or calcification. MRI is the only widely used functional imaging today using IV contrast to show increased flow associated with a mass or malignant structure. The higher sensitivity of MRI is in large part due to the use of contrast, which adds a functional element to the examination. Contrast-enhanced mammography has been described but is not widely used [41,42]. This was developed in an attempt to gain more information from a mammogram and to make contrast imaging of the breast more available and more affordable than MRI. However, contrast-enhanced mammography still has to contend with compression and distortion and still requires at least two separate views per side.

**Figure 5:**
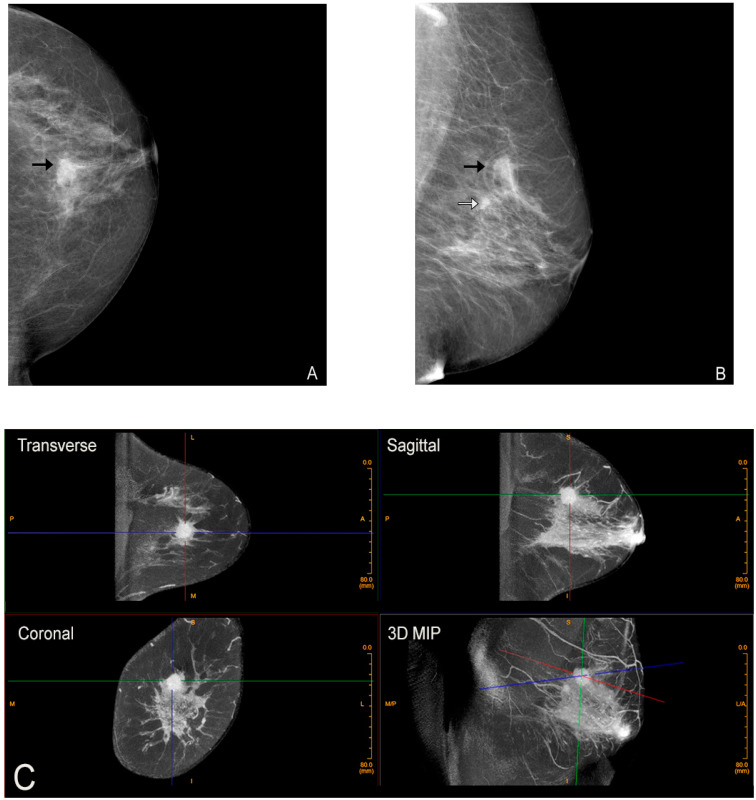

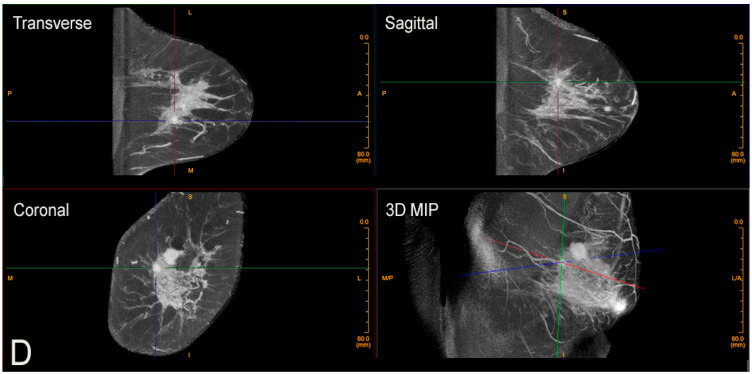
Contrast-enhanced CBBCT. Left CC (**A**) and MLO (**B**) views demonstrate two masses (black and white arrows). (**C**) Contrast-enhanced CBBCT with gridlines marking the mass corresponding to the black arrow on mammography. (**D**) Contrast-enhanced CBBCT with gridlines marking the mass corresponding to the white arrow on mammography.

CBBCT is easily performed after contrast administration (Figure 5). Each breast is imaged in a single 360-degree sweep, before and after contrast administration. Through a peripheral IV using a power injector, non-ionic iodine-based contrast is injected at a rate of 2 cc per second, 1 mg per kg, and the breast is imaged after a short delay (90 s) [29]. The other breast may then also be imaged within the next 10 min with excellent enhancement seen. With the use of IV contrast enhancement, the use of CBBCT is expanded to include many of the indications for contrast-enhanced MRI. These include evaluation of extent of disease after a cancer diagnosis, evaluation of response to neoadjuvant chemotherapy, and importantly, imaging women who have a contraindication to MRI such as pacemakers, implanted metallic devices, or claustrophobia. In addition, use of CBBCT circumvents concerns with serial administration of gadolinium and its deposition in the brain [43].

In conclusion, breast imagers must openly acknowledge the limitations of current technologies, which have been developed to address an urgent need to screen for breast cancer. With constant improvements over the past decades, there has been a demonstrated 40% reduction in breast cancer mortality for those actively screened [44,45,46]. However, we must admit we still fall short and cannot overcome the limitations of painful compression, the need for multiple views, and the inherent enemy of cancer detection, intrinsic breast density. It is time for a paradigm shift. CBBCT provides true 3D imaging in a single sweep without painful compression. This may bring us to a situation where we have fewer false positives and, more importantly, fewer false negatives. This is achieved by using true isotropic 3D imaging for morphological features, combined where indicated with IV contrast for functional imaging, thereby giving us maximum information for earlier cancer detection. Together with improved treatment options, this could lead to better outcomes for all women.

## Data Availability

Not applicable.

## Supplementary Materials

The following are available online at https://www.mdpi.com/article/10.3390/jcm10215135/s1, Video S1: Video demonstrating how cone-beam breast computed tomography (CBBCT) image acquisition occurs.
